# HPV‐Associated Adenosquamous Carcinoma of the Oropharynx With a *PARD3B* :: *ERBB4* Fusion

**DOI:** 10.1002/gcc.70165

**Published:** 2026-09-12

**Authors:** Seddig Momenkhan, Jacques Blanc, Pierre Philouze, Jonathan Lopez, Francoise Descotes, Eudeline Alix, Camille Leonce, Ziyad Alsugair, Nazim Benzerdjeb

**Affiliations:** ^1^ Service d'Anatomie et cytologie pathologiques Institut de Pathologie Multisite, Hospices Civils de Lyon, CHU Lyon Sud Pierre‐Bénite France; ^2^ Faculty of Medicine King Abdulaziz University Jeddah Saudi Arabia; ^3^ Service d'Oto‐Rhino‐Laryngologie et Chirurgie Cervico‐Faciale Hôpital La Croix Rousse, Hospices Civils de Lyon Lyon France; ^4^ Service de Biochimie et Biologie Moléculaire, CHU Lyon Sud, Hospices Civils de Lyon Université Claude Bernard Lyon I Pierre‐Bénite France; ^5^ Service d’Anatomie et cytologie pathologiques, Institut de Pathologie Multisite Hospices Civils de Lyon, CHU Lyon Est Pierre‐Bénite France; ^6^ CICLY Pierre‐Bénite France

**Keywords:** adenosquamous carcinoma, *ERBB4*, HPV, oropharynx, *PARD3B*

## Abstract

Head and neck carcinomas can be diagnostically challenging because of their diverse histologic subtypes. We report a case of a 55‐year‐old male presenting with right‐sided cervical adenopathy, in whom imaging revealed a cystic lesion that was subsequently confirmed to be metastatic from a lesion at the base of the tongue. Histological examination of the lesion at the base of the tongue demonstrated a biphasic tumor proliferation composed of nonkeratinized basaloid cells arranged in cohesive lobules, with focal small ductal structures. Immunohistochemistry highlighted the biphasic morphology, with strong nuclear expression of p40/p63 in the abluminal cells and prominent membranous staining of CEA and CK7 in the luminal cells. Ancillary testing further characterized the lesion, identifying an HPV16 by DNA PCR. RNA sequencing revealed a *PARD3B::ERBB4* fusion involving *PARD3B* exon 13 and *ERBB4* exon 17, while targeted next‐generation sequencing identified a *KRAS* exon 2 c.35G>A (p.Gly12Asp) mutation. Microdissection‐based analysis confirmed the same *KRAS* mutation in both the glandular and squamous components, supporting a shared clonal origin. This case highlights the clinicopathologic complexity of HPV‐associated adenosquamous carcinoma and expands the molecular spectrum of head and neck tumors through identification of a *PARD3B*::*ERBB4* fusion.

## 1 Introduction

Adenosquamous carcinoma (ASC) is a rare and aggressive malignancy defined by the coexistence of malignant squamous and glandular components within the same tumor. Histologically, it consists of invasive squamous cell carcinoma associated with true adenocarcinoma demonstrating gland formation and/or intracellular mucin production [1]. ASC has been described in several anatomical sites, including the lung, pancreas, uterine cervix, and upper aerodigestive tract [2]. In the head and neck region, ASC behaves as a high‐grade carcinoma characterized by aggressive local invasion and frequent nodal metastasis. However, the molecular mechanisms underlying its biphasic differentiation remain poorly understood.

High‐risk human papillomavirus (HPV) infection has been implicated in a subset of ASCs arising in the oropharynx and sinonasal tract. Transcriptionally active HPV has been detected in approximately 16% of cases and appears to be associated with improved clinical outcomes compared with HPV‐negative tumors [1]. Nevertheless, beyond viral oncogenesis, the genomic drivers contributing to ASC tumorigenesis remain largely undefined.

The *ERBB4* gene, located on chromosome 2q34, encodes a member of the ERBB family of receptor tyrosine kinases involved in epithelial proliferation and differentiation. ERBB4 signaling plays context‐dependent roles in cancer biology [3, 4], and dysregulation of ERBB pathway components has been associated with aggressive behavior in squamous malignancies [3, 4]. However, *ERBB4* alterations have not been reported in head and neck ASC. Here, we report a case of HPV‐associated head and neck ASC with comprehensive molecular profiling, identifying a novel *ERBB4* alteration and discussing its biological significance.

## 2 Case

### 2.1 Clinical Presentation

A 55‐year‐old male patient presented with right‐sided cervical adenopathy. Ultrasound evaluation illustrated a cystic lesion. MRI confirmed the cystic characteristics of the mass without identifying additional lesions. The patient underwent surgical excision of the mass along with ipsilateral cervical lymph node dissection. A diagnosis of intermediate‐grade mucoepidermoid carcinoma was made by the initial pathologist. Further investigation with upper panendoscopy revealed no obvious lesions, but subtle changes at the base of the tongue prompted a biopsy. The case was discussed in a multidisciplinary tumor board, and the decision was made to treat the patient with exclusive adjuvant local radiotherapy.

### 2.2 Histology and Immunohistochemistry

**FIGURE 1:**
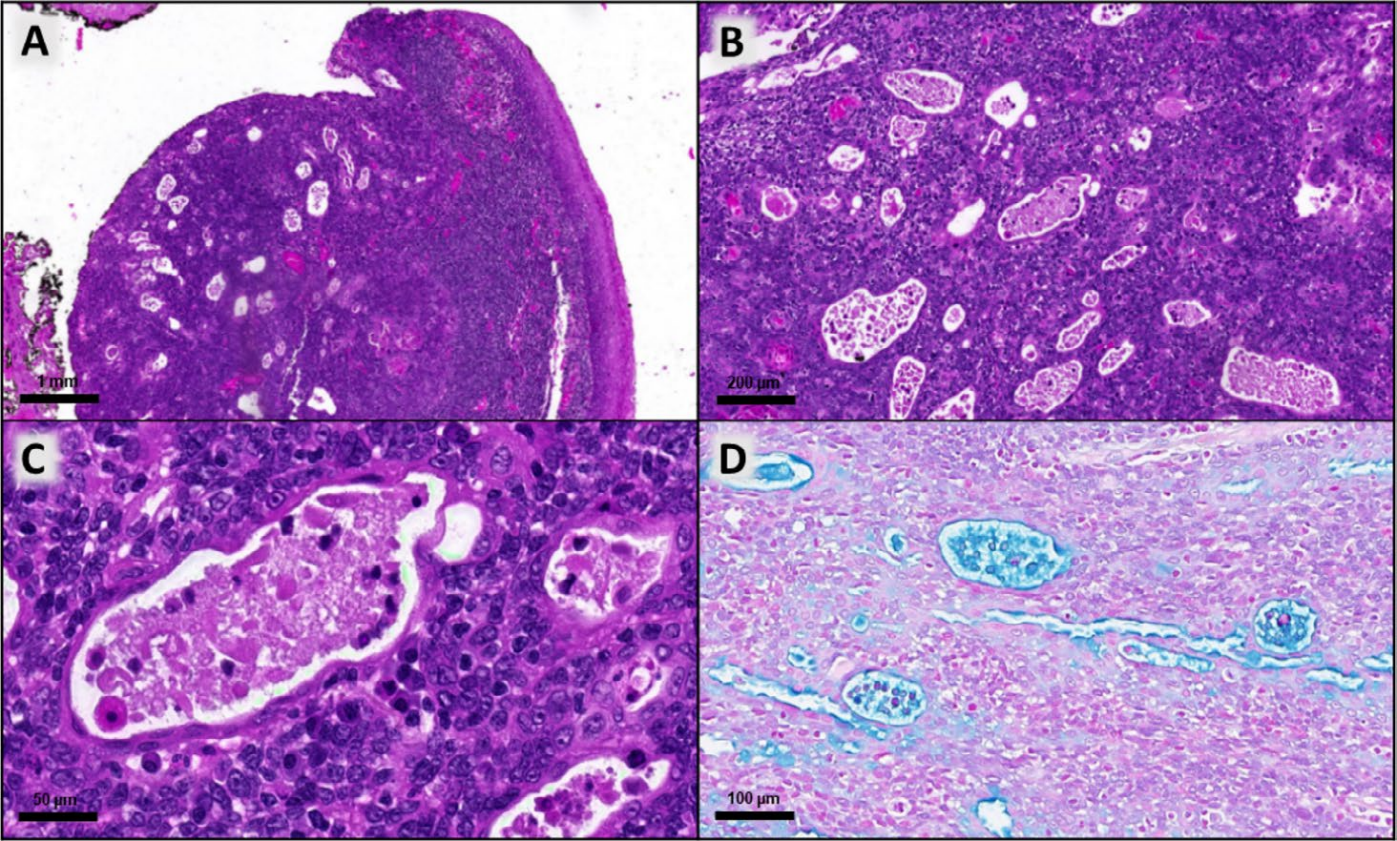
(A, ×2 magnification) Low‐power view of the base‐of‐tongue lesion demonstrating nondysplastic surface epithelium overlying crypts with an underlying basaloid tumor proliferation and prominent lymphoid germinal centers. (B, ×10 magnification) Higher magnification reveals small duct‐like structures within the tumor. (C, ×40 magnification) The tumor is composed of cohesive lobules of cells displaying a high nuclear‐to‐cytoplasmic ratio and focal central necrosis. (D, ×20 magnification) Alcian blue staining highlights the luminal aspect of the ductal component, without identifiable mucin‐producing cells.

Histological examination of the lesion from the base of the tongue revealed a tumor proliferation (Figure 1A). The crypts exhibited a notable nonkeratinizing basaloid cell proliferation arranged in cohesive lobules. A distinctive feature of small ductal structures was observed focally (Figure 1B). The tumor cells were characterized by a high nuclear‐to‐cytoplasmic ratio and occasional central necrosis (Figure 1C). Alcian blue staining highlighted the luminal ductal component, without identifiable mucin‐producing cells (Figure 1D). Histologic examination of the cystic cervical mass confirmed a lymph node metastasis. No significant morphologic differences were observed between the metastatic lesion and the primary base‐of‐tongue tumor; both showed the same biphasic squamous and glandular architecture.

**FIGURE 2:**
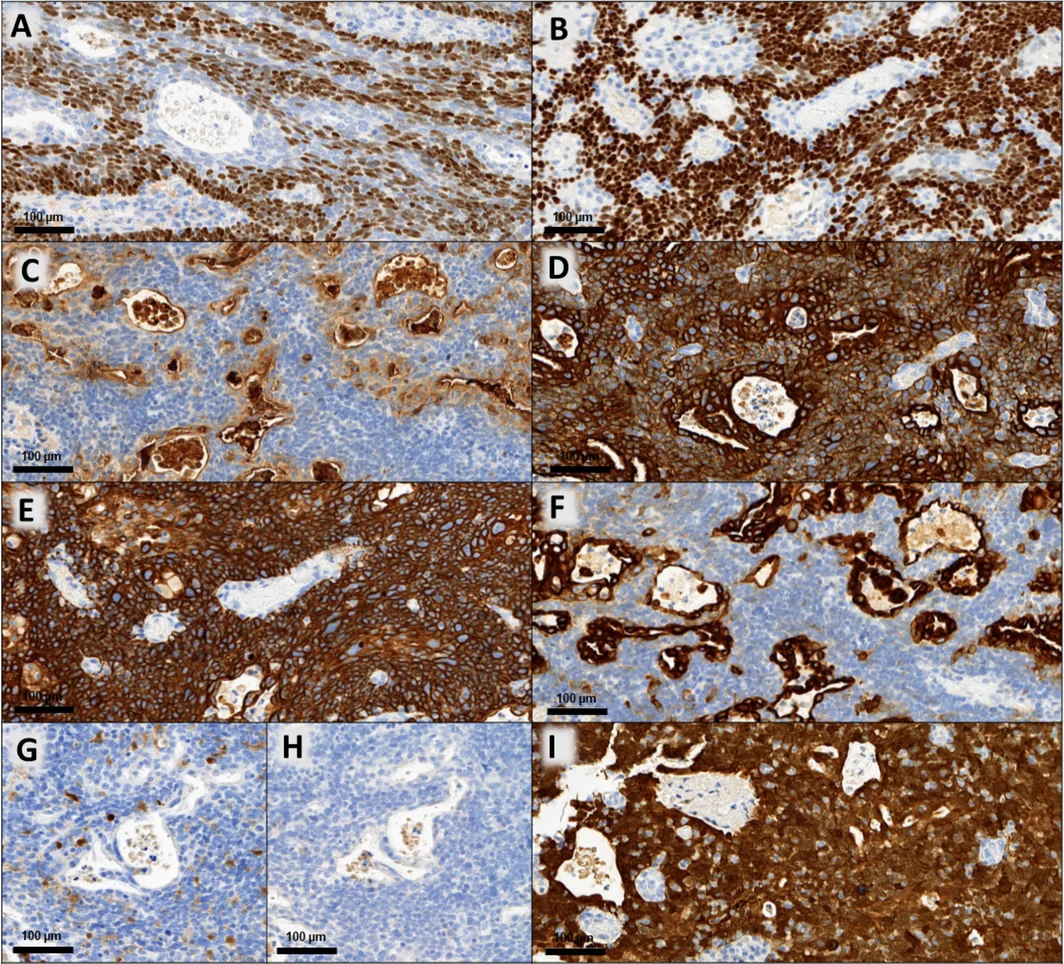
(A, ×20 magnification) p40 demonstrates strong nuclear expression in abluminal cells; (B, ×20 magnification) p63 reveals strong nuclear expression in abluminal cells; (C, ×20 magnification) CEA shows strong membranous positivity in luminal cells; (D, ×20 magnification) CK7 displays biphasic membranous expression, with stronger staining in luminal cells and milder staining in abluminal cells; (E, ×20 magnification) CK5/6 exhibits intense membranous staining in abluminal cells; (F, ×20 magnification) EMA shows prominent membranous expression in luminal cells, with weaker staining in abluminal cells; (G and H, ×20 magnification) S100 and SOX10, respectively, are negative; and (I, ×20 magnification) p16 demonstrates marked nuclear and cytoplasmic positivity in tumor cells.

Immunohistochemistry highlighted the biphasic nature of the tumor in which p40/p63 showed strong nuclear expression in the tumor's abluminal cells (Figure 2A,B), while CEA showed strong membranous expression in the tumor's luminal cells (Figure 2C). CK7 displayed a biphasic expression, with strong staining in the luminal cells of the ducts and milder expression in the tumor's abluminal cells (Figure 2D). CK5/6 demonstrated strong membranous expression in abluminal cells (Figure 2E). EMA exhibited strong membranous expression in the luminal cells (Figure 2F). Both S100 and SOX10 were negative (Figure 2G,H). p53 displayed a wild‐type expression pattern, characterized by scattered weak nuclear staining. p16 showed marked nuclear and cytoplasmic overexpression in more than 70% of the tumor cells (Figure 2I). Overall, the biphasic morphology (squamous and glandular differentiation), immunophenotype, and HPV16 positivity supported a diagnosis of HPV‐associated ASC of the oropharynx.

### 2.3 Molecular Studies

**FIGURE 3:**
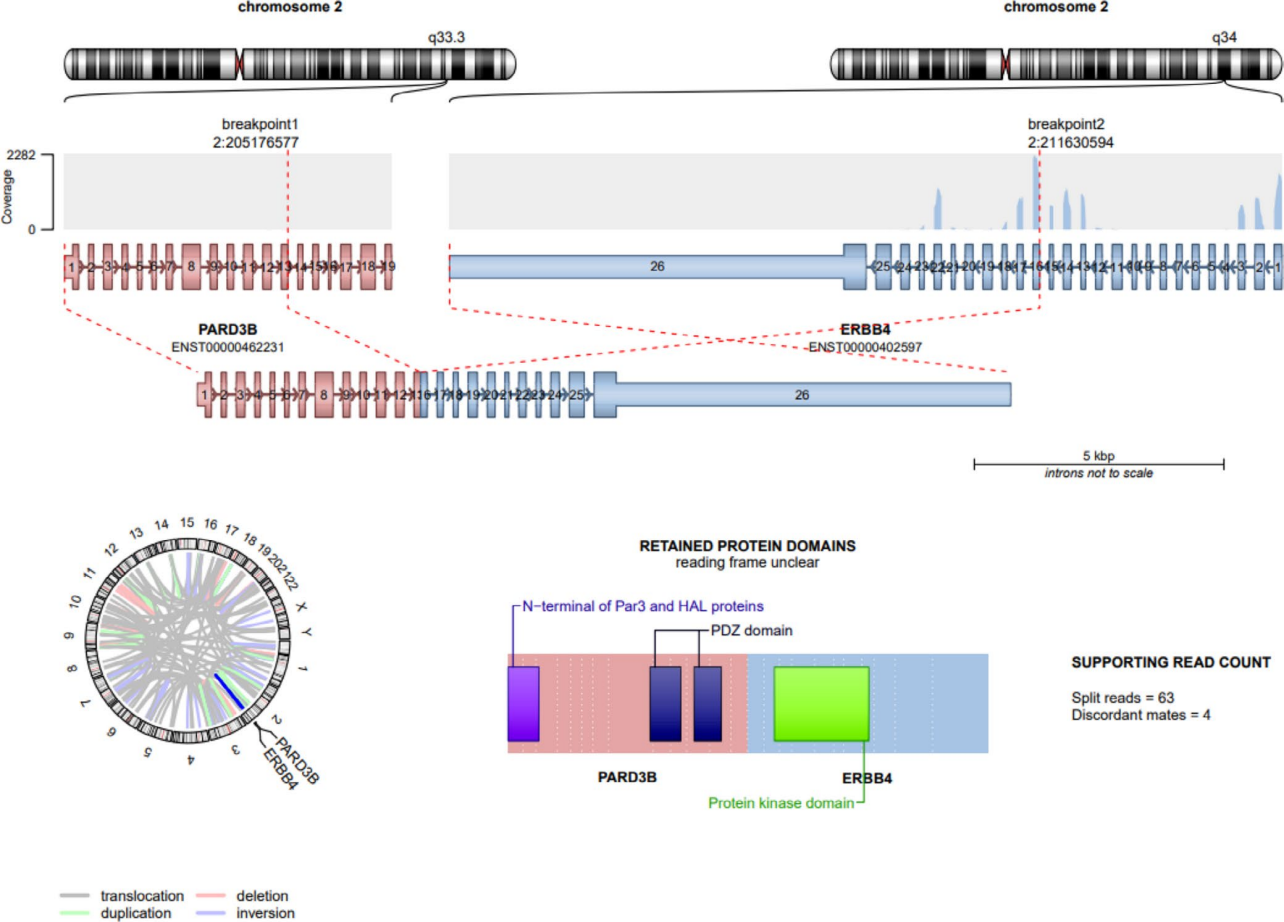
Schematic representation of the *PARD3B*::*ERBB4* fusion (breakpoints at *PARD3B* exon 13 and *ERBB4* exon 17) detected by RNA sequencing.

**FIGURE 4:**
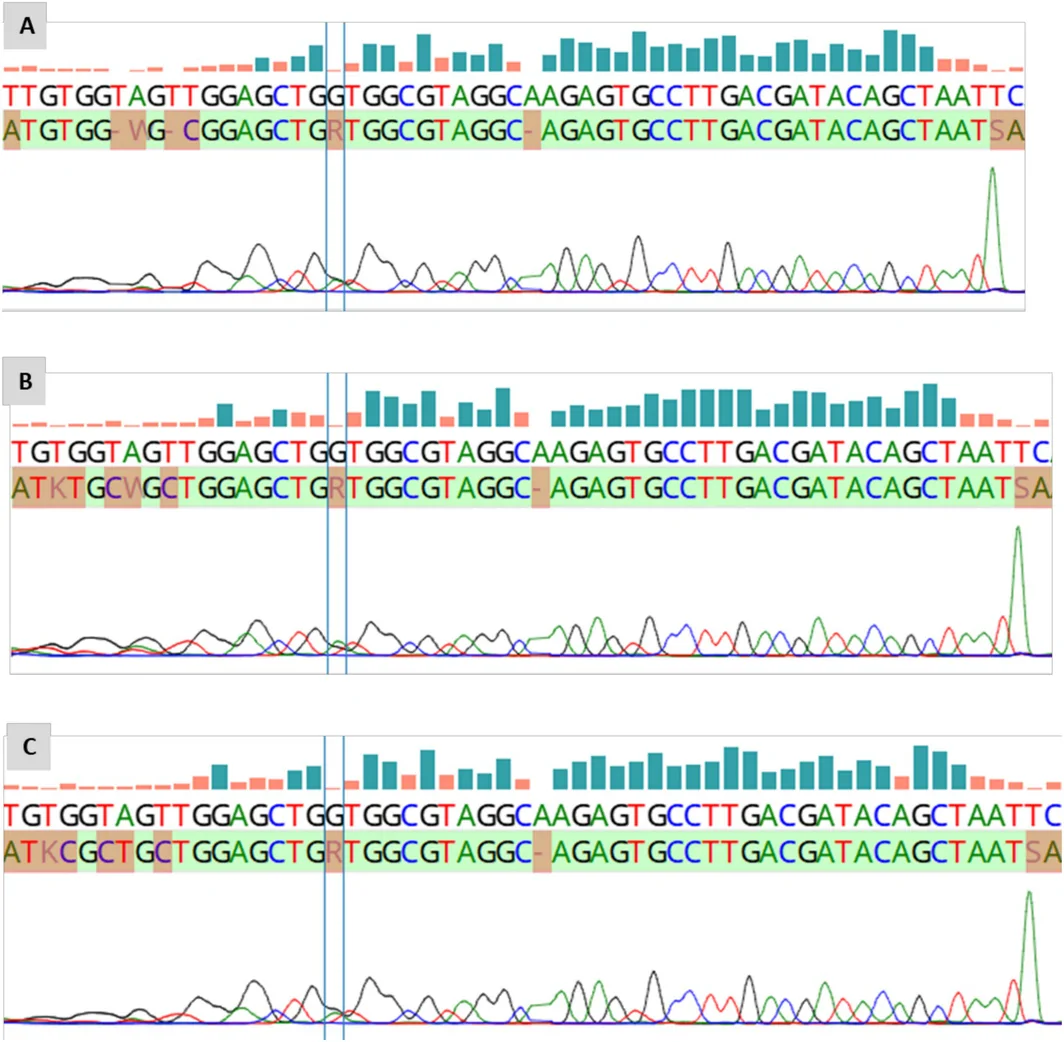
Component‐specific analysis of the *KRAS* mutation after microdissection. Sequence analysis demonstrates the *KRAS* c.35G>A (p.Gly12Asp) mutation in the microdissected glandular component (A), the microdissected squamous component (B), and the initial whole‐tumor sample (C). The identical alteration in both morphologic components supports a shared clonal origin.

For further characterization of the tumor, molecular genotyping performed using DNA PCR (Inno‐LiPA HPV) identified an HPV16 variant. RNA sequencing was conducted using the Custom RNA Target Bundle Solution (Sophia Genetics) on an Illumina NextSeq 500 platform. This analysis detected a *PARD3B*::*ERBB4* fusion, with breakpoints at *PARD3B* exon 13 and *ERBB4* exon 17 (Figure 3). This fusion was supported by 63 split reads and four discordant mate pairs and retained the ERBB4 protein kinase domain. However, orthogonal validation was not performed, and the functional significance of this fusion remains to be established. Targeted next‐generation sequencing (TNGS), performed using a 107‐gene custom panel from Sophia Genetics on an Illumina NextSeq 500 platform, revealed a *KRAS* exon 2 c.35G>A (p.Gly12Asp) mutation. No *MAML2, DEK, PLAG1*, *HMGA2*, *MYB*, *MYBL1*, or *FGFR3* fusion was detected by RNA sequencing, and no pathogenic alteration was identified in *TP53* or *PIK3CA* by targeted DNA sequencing. To investigate the distribution of this alteration, the glandular and squamous components were separately microdissected and analyzed. The same *KRAS* c.35G>A (p.Gly12Asp) mutation was detected in both components and was identical to that identified in the initial whole‐tumor analysis (Figure 4). Component‐specific RT‐PCR analysis of the *PARD3B::ERBB4* fusion was also attempted. However, despite microdissection of substantial tumor areas, the quantity and quality of the recovered RNA were insufficient for reliable amplification and sequence interpretation. Therefore, the distribution of the fusion between the two components could not be determined.

## 3 Discussion

We report a case of HPV‐associated ASC harboring a *PARD3B*::*ERBB4* fusion. This tumor presents a potential diagnostic pitfall due to its biphasic morphology, characterized by tubular structures and a broad luminal proliferation. Immunohistochemistry with CK7 and CEA in the luminal component, together with p40/p63 in the abluminal component, highlights this biphasic differentiation. The identification of the same *KRAS* c.35G>A (p.Gly12Asp) mutation in both the glandular and squamous components supports a common clonal origin of the biphasic tumor. In contrast, the spatial distribution of the *PARD3B::ERBB4* fusion could not be determined because component‐specific RT‐PCR analysis was technically inconclusive owing to the limited quantity and suboptimal quality of RNA recovered after microdissection. Initially, the metastatic mass was interpreted as mucoepidermoid carcinoma by the referring pathologist because of its biphasic architecture and focal ductal differentiation. However, upon further evaluation at our specialized center in head and neck pathology, a panendoscopy revealed the primary lesion, confirming the metastatic origin of the cervical mass. This initial misdiagnosis is not surprising, as biphasic morphology is a well‐documented feature of several salivary gland neoplasms, including adenoid cystic carcinoma, mucoepidermoid carcinoma, and epithelial–myoepithelial carcinoma [5, 6, 7]. However, integration of the oropharyngeal primary site, diffuse p16 overexpression with HPV16 detection, and negative SOX10/S100 favored HPV‐associated ASC over primary salivary gland carcinoma, including mucoepidermoid carcinoma. HPV association was supported by diffuse p16 overexpression and HPV16 DNA positivity. However, HPV16 E6/E7 transcript expression was not assessed; therefore, transcriptionally active HPV infection could not be directly confirmed. This combination of the biphasic morphology and the HPV positivity has been also reported in various head and neck tumors, as well as in salivary gland neoplasms. These include squamous cell carcinoma, ASC, mucoepidermoid carcinoma, acinic cell carcinoma, and adenoid cystic carcinoma along with the most recently recognized entity, HPV‐related multiphenotypic sinonasal carcinoma, which exhibits a squamous dysplasia and a salivary component mimicking the solid variant of adenoid cystic carcinoma [8]. In the present study, the histological features are similar to those of HPV‐related multiphenotypic sinonasal carcinoma. However, it harbored an HPV genotype 16 rather than genotype 33, and arose in the base of the tongue rather than the sinus [9]. Nevertheless, it was important to rule out other diagnoses through molecular biology due to the existence of numerous overlapping diagnoses in head and neck pathology [10]. Recently, several fusions were identified in the oropharynx such as *FGFR3*‐associated or not with HPV [11]. In head and neck, carcinomas harboring *FGFR3*::*TACC3* display a significant heterogeneity in terms of morphology. These carcinomas have been observed in three distinct prototypes: (i) unclassified carcinomas with distinct morphological features that suggest a new tumor type driven by the fusion, (ii) HPV‐associated carcinomas (mainly sinonasal) with concurrent *FGFR3*::*TACC3* fusions, and (iii) conventional squamous cell carcinomas [12]. It is important to note that while HPV infection is a known risk factor for the development of head and neck squamous cell carcinoma (HNSCC), other factors such as gene alterations play roles in the development of this type of cancer [13]. Additionally, studies pointed to the role of gene fusions as a transforming event in HPV‐related carcinogenesis. Of note, the activation of the FGFR pathway has been postulated to play a central role in HPV‐associated cervical oncogenesis [14, 15].

The *ERBB4* gene, located at 2q34, is a member of the *ERBB* family of receptor tyrosine kinases, which plays a crucial role in cellular signaling, differentiation, and survival. This family includes *EGFR* (*ERBB1*), *ERBB2* (*HER2*), *ERBB3 (HER3)*, and *ERBB4* (*HER4*) [3, 4]. They have been involved in cancer progression, as well as in mechanisms regulating epithelial‐to‐mesenchymal transition, through interactions with various proteins such as TGF β or SMAD4 [3, 16]. *ERBB* family fusions are recurrent molecular alterations identified as actionable targets across various cancer types, with an incidence approaching 0.7% [17]. *ERBB4* fusions have been previously reported in ovarian cancer, lung adenocarcinoma, and colorectal cancer [18, 19]. It has also been identified in head and neck tumors as the *ATAD2*::*ERBB4* fusion in a case of poorly to moderately differentiated squamous cell carcinoma of the larynx [20, 21].

*PARD3B*, located at 2q33.3, is a member of the Par‐3 family of polarity proteins and is involved in epithelial cell adhesion and polarity. In addition to being a scaffolding protein, it is linked to basal layer stem cells and cadherin‐Src signaling, thereby contributing to the induction of epithelial‐to‐mesenchymal transition [22, 23, 24]. Multiple studies have implicated that *PARD3B* deregulation disturbs the balance between asymmetric and symmetric cell division, promoting neoplastic transformation through uncontrolled self‐renewal of cancer cells and tumor‐initiating cells [24]. *PARD3B* fusions have been documented in various types of cancer such as breast carcinomas, colorectal adenocarcinomas, liposarcomas, and lung cancers. In the head and neck region, the *NRP2*::*PARD3B* fusion has been reported twice. Specifically, in two cases of invasive squamous cell carcinoma [20, 21]. *PARD3B::ERBB4* fusion has been described in limited reports: once as an antisense fusion in colorectal adenocarcinoma, while details of a second reported case were unavailable at the time of writing [17, 20, 21].

HNSCC comprises two biologically distinct entities defined by HPV status, each with a characteristic mutational profile. HPV‐negative, smoking‐related tumors are characterized by frequent loss‐of‐function *TP53* mutations and *CDKN2A* inactivation, together with recurrent copy‐number gains at 3q26/28 and 11q13/22 [25]. HPV‐associated tumors, in contrast, generally retain wild‐type *TP53*, with *p53* function disrupted by E6 and are enriched in helical‐domain *PIK3CA* mutations, *TRAF3* loss, and *E2F1* amplification [25]. Other recurrently altered genes across HNSCC include *NOTCH1*, *FAT1*, *AJUBA*, *CASP8*, *HRAS*, *NSD1*, and *NFE2L2* [25, 26, 27], with *NOTCH1* behaving predominantly as a tumor suppressor in this tumor type [26, 27]. Overall, HPV‐negative tumors carry a higher mutational burden than HPV‐positive tumors [13, 27]. Situating the alterations observed in the present case within this framework helps clarify whether they fall within the expected genomic landscape of HNSCC or represent an uncommon finding.

## 4 Conclusion

HPV‐associated ASC harboring *PARD3B*::*ERBB4* expands the molecular spectrum of head and neck tumors and underscores the importance of integrated morphologic, immunohistochemical, and molecular assessment in biphasic oropharyngeal carcinomas.

## Author Contributions

S.M. and N.B. designed the study; S.M., Z.A., and N.B. reviewed the case; J.B. and P.P. provided the clinical data; J.L. and F.D. performed the molecular analysis; S.M. and N.B. wrote the manuscript; S.M., N.B., Z.A., and J.B. revised the article. E.A. was involved in the RT‐PCR attempt. C.L. was involved in the microdissection.

## Funding

The authors have nothing to report.

## Ethics Statement

All procedures performed in studies involving human participants were in accordance with the ethical standards of the institutional and/or national research committee and with the 1964 Helsinki Declaration and its later amendments, or comparable ethical standards.

## Consent

Informed consent was obtained from the individual participant included in the study.

## Conflicts of Interest

The authors declare no conflicts of interest.

## Data Availability

The data that support the findings of this study are available on request from the corresponding author. The data are not publicly available due to privacy or ethical restrictions.
